# The reliability of posterior malleolar ankle fracture assessment: a unique perspective

**DOI:** 10.1007/s00590-023-03702-y

**Published:** 2023-08-29

**Authors:** Edward Joseph Fűzy, Nando Ferreira, Craig Brown, Daniel Hugo, Etienne Joubert, Marilize Burger

**Affiliations:** Division of Orthopaedic Surgery, Department of Surgical Sciences, Faculty of Medicine and Health Sciences, Stellenbosch University, Cape Town, 7505 South Africa

**Keywords:** Posterior malleolus fracture, Trimalleolar fracture, Posterior tibial rim, Ankle fracture, Surgical fixation, Classifications

## Abstract

**Aim:**

This study aims to elucidate the pathology of PMFs in the South African population, establish correlations between fracture patterns and international classification guidelines and evaluate the interobserver reliability of current classifications.

**Methods:**

A retrospective review was conducted in a multicentre analysis over a one-year period from January 2019 to December 2019 at our institution. Computer tomography scans for foot and ankle injuries were reviewed, and posterior malleolus fractures were included. Pathoanatomical data was collected and analysed according to known classification systems and subsequent treatment modalities evaluated. A panel of observers individually reviewed radiographic data to determine interobserver reliability.

**Results:**

A total of 71 patients were included with a mean age of 41 ± 13.4 years (range 18–78) and a female predominant population (69%). A greater proportion of injuries were high energy (23.9%), with significant fragment comminution (53.5%), and half (52.1%) of all injuries were subluxated/dislocated at presentation. A total of 93% of injuries were managed operatively, despite theatre access limitations resulting in significant delays to fixation (19.1 days). Despite good pathoanatomical agreement with most international classifications, interobserver reliability was poor (Krippendorff *α*-coefficient < 0.667). Inconsistent treatment patterns in operative and non-operative strategies are reported.

**Conclusion:**

A unique patient population of younger, female individuals incurred posterior malleolar fractures due to higher energy mechanisms of injury. Whilst injury patterns were mostly comparable, significant interobserver variability was noted. Resource limitations, diagnostic challenges, poorly defined and inconsistent treatment strategies, inevitably impact outcomes within the South African population.

**Level of evidence:**

Level III.

## Introduction

Posterior malleolus ankle fractures (PMF) pose ongoing controversies in orthopaedic literature and clinical practice [1, 2]. Despite varying incidences ranging from 7 to 46%, the specific characteristics of these injuries in the African continent remain poorly quantified [1, 3–6]. Biomechanically, the posterior malleolus plays a crucial role in maintaining syndesmotic stability and ensuring uniform tibiotalar contact stressors [7, 8]. Injury to the posterior malleolus typically occurs through low-velocity, twisting-type mechanisms, as indicated by the Lauge-Hansen classification, which signifies the systematic and progressive disruption of key ankle restraints [3, 9, 10]. Consequently, joint congruency and stability are compromised within the tibiotalar joint and syndesmosis [11].

Previous studies have reported improved clinical outcomes with surgical repair of the posterior malleolus when more than 25% of the articular surface was involved, establishing a controversial size-based threshold for surgical intervention [12]. However, recent literature highlights the significance of joint congruity and syndesmotic stability as critical factors in treatment planning [2, 13]. Computed tomography (CT) scanning has emerged as the gold standard for pre-operative evaluation of PMFs, owing to the unreliable and inaccurate interpretation of plain film radiographs [14–16]. CT scans guide the preferred surgical approach and enhance direct fragment fixation method [17]. Various systemic classification systems by Haraguchi et al., Bartonicek et al., Mason et al. and Lu et al., based on 2D axial CT cuts have been proposed, but their interpretation lacks consensus regarding optimal management and reliability [9, 11, 18–22].

Treatment options for PMFs exhibit variability, with recent biomechanical studies favouring posterior fragment-specific fixation over the commonly performed anteroposterior (AP) screw fixation [9, 21]. However, posterior fragment-specific fixation presents challenges, including increased surgical complexity, a higher risk of malreduction, elevated failure rates, and poorer outcomes [8, 9, 20–26]. The prevalence of post-traumatic osteoarthritis following PMFs, which affects 33.5% of cases, leads to substantial disability contingent upon the restoration of tibiotalar congruency, contact stressors, and syndesmotic stability [2, 5, 11]. Inconsistent classification systems, evaluation methods, surgical thresholds, and techniques further complicate outcome interpretation.

This study aims to elucidate the pathology of PMFs in the South African population, establish correlations between fracture patterns and international classification guidelines, and evaluate the interobserver reliability of current classifications. By addressing these objectives, the study seeks to enhance our understanding of PMFs, provide valuable insights into their management and guide treatment decisions in the South African context.

## Methods

This retrospective study reviewed the records of all patients with ankle fractures involving the posterior malleolus between January 2019 and December 2019 at three public centres, including a tertiary, district and regional hospitals. Institutional ethics committee and hospital board approval were obtained prior to data collection.

**Fig. 1:**
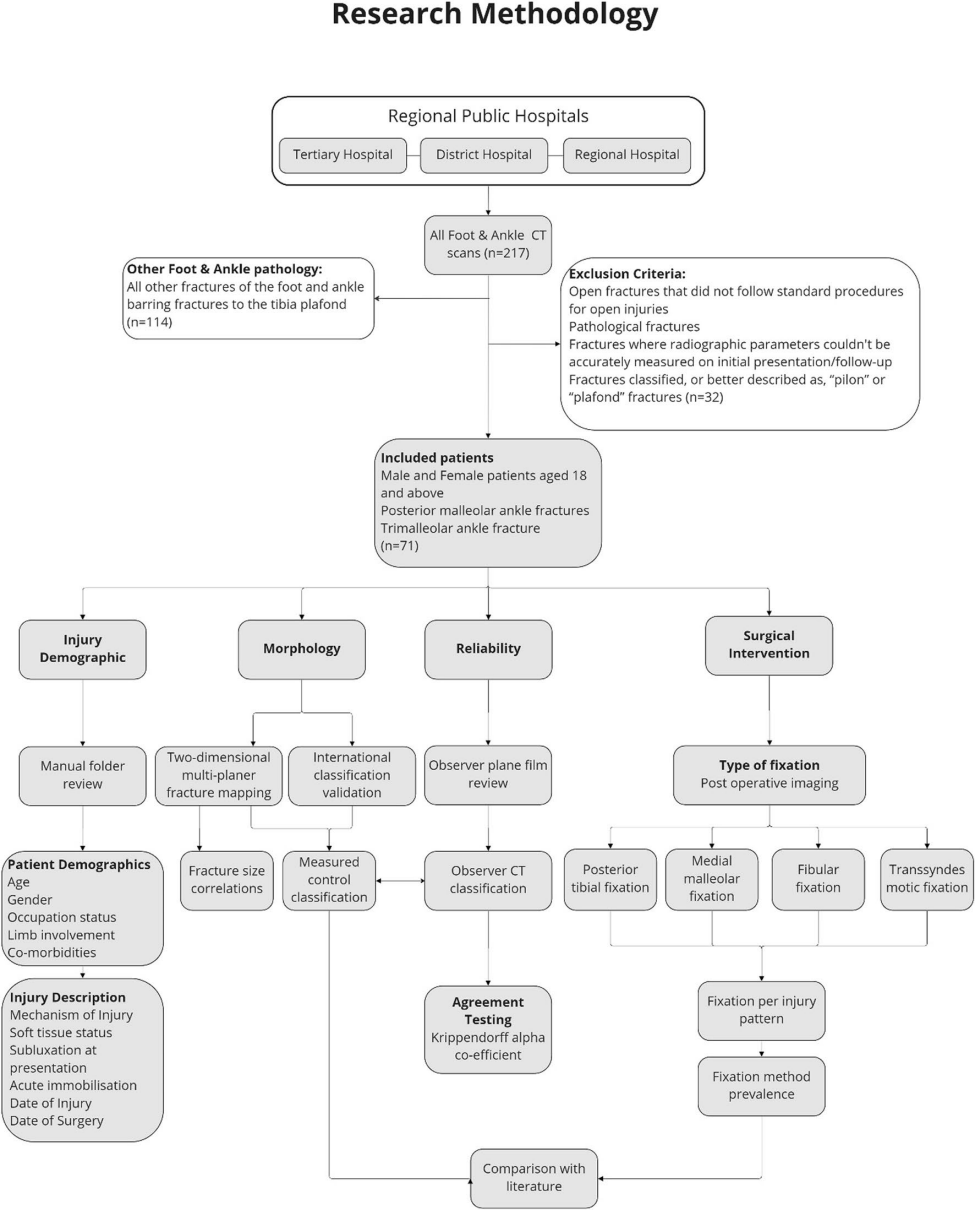
Detailed methodology process flow of all participants reviewed in the posterior malleolus fracture study over a one-year period

All adult patients who underwent foot and ankle computed tomography scans during the study period were considered for inclusion. Patients with posterior malleolar or trimalleolar ankle fractures were included (Fig. 1). Data were collected regarding patient demographics, injury characteristics, radiological evaluation and treatment.

The above flow diagram represents the inclusion and exclusion process of 217 CT scans performed in a one-year period for variable foot and ankle pathology. After exclusion, 71 patients remained eligible for further examination. Four distinct processes were followed, namely, i) establishing the patient and injury descriptive data; ii) mapping the fracture patterns and measuring fracture variables to establish data points that were used to compare with known classification data points; iii) fractures were classified on CT imaging and interobserver reliability testing performed, in addition to comparing clinical observations to measured data points (*measured control classification*) and iv) surgical fixation methods reviewed on fluoroscopic imaging, then compared to individual classification types and to international surgical fixation rates and methods.

**Fig. 2:**
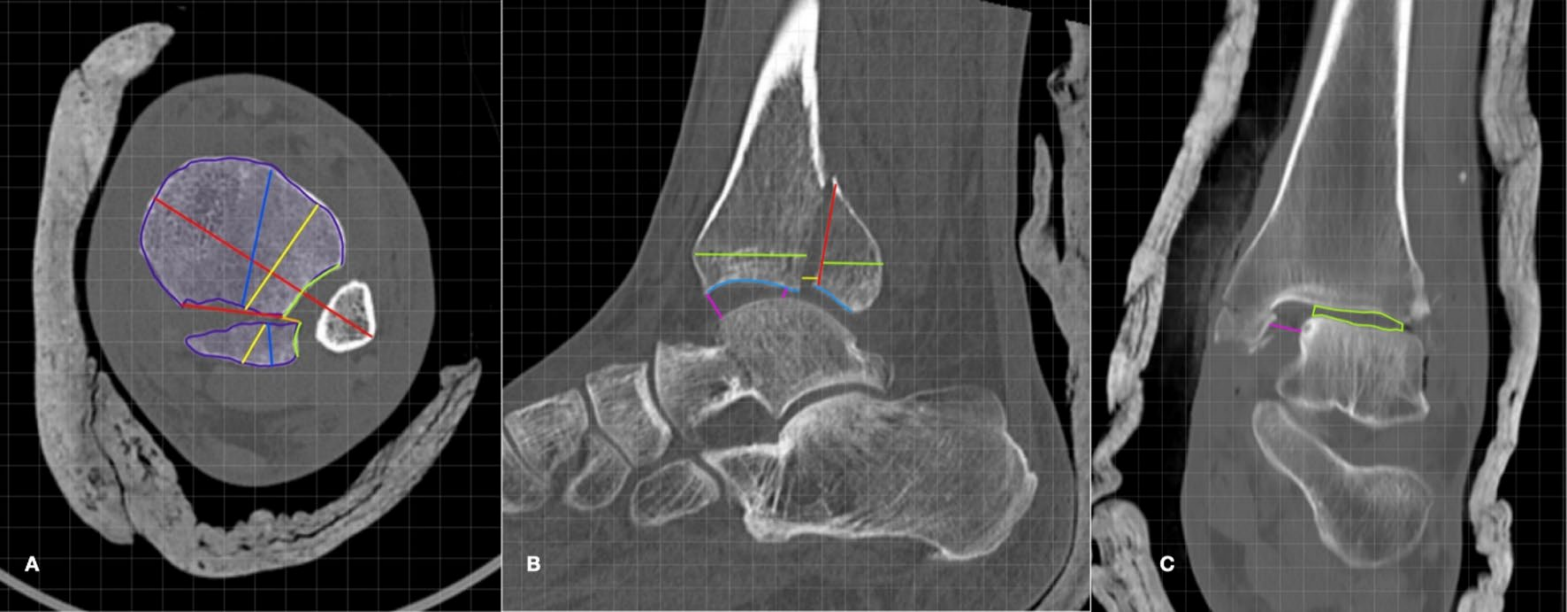
Examples of the fracture mapping done on all posterior malleolus fractures in a multicentre study over a one-year period (color figure online)

Standard imaging protocols with one millimetre (fine slice) CT images were evaluated in the coronal, sagittal and axial planes (standard 3 mm above the joint line). Sagittal films were examined in the slice that revealed the greatest cross-sectional area of the posterior malleolus fragment, and coronal images sectioned at the trans-malleolar line. No 3D reconstructions were examined. Individual fracture morphology was mapped by importing CT images into SketchAndCalc online software and analysed according to size, shape, fracture pattern, angle of primary and secondary fracture lines, overall plafond area–sagittal and axial, fracture fragment area–sagittal and axial, incisural extension, any noted comminution, displacement of the fragment and widening of the talofibular clear space (Fig. 2). The exact parameters required to fulfil the criteria of each classification system were measured to establish an objective data set (*Control Classification*), used as a control in further comparisons.

Figure 2A Axial Computer Tomography (CT) scan images taken 3 mm above the joint line. Examples in this image show (*purple*) the area measurement of the plafond and the fragment; (*blue*) the axial length measurement of the fragment, perpendicular to the fracture line; (*yellow*) the axial length measurement of the fragment, perpendicular to the trans-malleolar axis; (*orange*) the displacement of the fragment, in relation to the tibiofibular clear space; (*green*) the proportion of incisural joint surface attached to the fracture fragment; and (*red*) the primary fracture angle, as measured from the trans-syndesmotic plane.

Figure 2B Mid-sagittal CT scan images depicting the fracture fragment. Examples in this image show (*red*) the height of the fracture fragment, at the fracture line; (*yellow*) the amount of anteroposterior (AP) displacement; (*green*) the sagittal length measurement of the fragment, in the direct AP plane; (*blue*) the sagittal length measurement of the fracture fragment, along the tibiotalar joint surface; and (*purple*) the joint space was measured at multiple intervals, inconsistencies with varying lengths, denoting sagittal tibiotalar joint subluxation.

Figure 2C Mid-coronal CT scan images depicting medial malleolar fractures and coronal tibiotalar incongruity. Examples in this image show (*purple*) the medial tibiotalar clear space; and (*green*) the incongruity of the tibial and talar joint surfaces, denoting coronal tibiotalar subluxation.

Images are for descriptive purposes only. Actual measurements were measured using computer software (SketchandCalc), calibrated to reference parameters utilizing a digital grid overlay and standardized across the series.

The primary researcher and four orthopaedic trauma specialists with a minimum of six years’ experience analysed and classified all the images independently. Injuries were classified according to the Dennis-Weber [27], Lauge-Hansen [10, 28] and Association of Osteosynthesis (AO) [29] classifications for plain film radiographs and the Haraguchi [18], Mason [19] and Bartonicek [9] classifications for CT images. Plain film radiographs were utilized as preliminary imaging, aiding in the assessment of CT images and classifications upon which further analysis and comparison were conducted. Finally, intraoperative fluoroscopic images were reviewed, and the fixation methods were recorded for the medial malleolus, posterior malleolus, syndesmosis and fibula.

Data were analysed using Stata v15.1 software and are described as means ± standard deviations or medians (interquartile ranges, IQR), depending on distribution, or as frequencies (counts). Normality was assessed using the Shapiro–Wilk test, and the Spearman’s Rank correlation was utilized to investigate correlations between continuous data. The Spearman’s Rank coefficient (Rho or *ρ*) was interpreted as < 0.10 being a negligible correlation, 0.10–0.3 being a weak correlation, 0.40–0.69 being a moderate correlation, 0.70–0.89 being a strong correlation and 0.90–1.00 being a very strong correlation [30]. Differences between classifications in the present study and the original work of Bartonicek, Haraguchi and Mason were investigated with a Chi-square test.

Agreement between observers against the control measurement is reported as percentage agreement, whilst the level of agreement on the classification of the fracture patterns between the five raters was measured using Krippendorff’s alpha statistic, using the methods described by Klein [31, 32]. An alpha level of > 0.8 was classified as a “high” level of agreement, whilst a level < 0.667 was regarded as “no agreement”. Levels between 0.667 and 0.800 were considered ‘tentative agreement’. These conservative cutoff values were based on the recommendations of Krippendorf for the interpretation of values that influence important decision-making processes, such as medical interventions [33].

## Results

Over the one-year study period, 217 patients underwent CT scans for injuries to the foot and ankle. Of these, 114 patients were excluded, with injuries to other structures within the foot and ankle. Additionally, 32 patients were excluded as the injuries were better described as “pilon” or “plafond” fractures. (Table 1) The final cohort comprised 71 patients, with a female predominance (*n* = 49, 69%) and a mean age of 40.9 ± 13.4 years (range 18–78). Most patients were unemployed (*n* = 46, 64.8%). A limb predominance was noted, with left ankle fractures being dominant in the study cohort (*n* = 52, 73.2%). In total, 35 patients (49.5%) had no co-morbidities, whilst 22 patients (31%) had only one, and 14 patients (19.7%) had two or more.

**Table 1:** Patient demographics of all participants with posterior malleolus fractures in a multicentre study over a one-year study period

Characteristic	*N* = 71
Age (years)	
	40.9 ± 13.4
*Gender*	
Male	31.0 (22)
Female	69.0 (49)
*Occupation status*	
Employed	26.8 (19)
Unemployed	64.8 (46)
Pensioner	8.5 (6)
*Limb involvement*	
Left	73.2 (52)
Right	26.8 (19)
*Co-morbidities*	
Nil	49.3 (35)
1	31.0 (22)
≥ 2	19.7 (14)

Data are described as mean ± standard deviation as frequencies with counts in parentheses

The predominant mechanism of injury was low-velocity falls (*n* = 47, 66.2%), followed by pedestrian-vehicle accidents (*n* = 17, 23.9%) and assault (*n* = 2, 2.8%) (Table 2). Four patients (5.6%) sustained open injuries. Posterior subluxation was seen in 31 patients (43.7%), with mediolateral subluxation noted in 37 (52.1%). Four patients (5.6%) required temporary external fixation to maintain reduction of the ankle joint. The mean period from injury to definitive fixation was 19.1 days (IQR, 1–45). Contributory factors to this delay included limited theatre availability, poor soft tissue condition, inter-facility transfers and pre-operative medical optimization. Two patients (2.8%) sustained isolated posterior malleolar injuries. Fibula fractures occurred in 67 patients (94.4%), and the medial malleolus required fixation in 59 cases (83.1%).

**Table 2:** Injury description of all participants with posterior malleolus fractures in a multicentre study over a one-year study period

Description	*N* = 71
*MOI*	
Low-velocity fall	66.20 (47)
Pedestrian-vehicle accident	23.90 (17)
Assault	2.80 (2)
Other*	7.10 (5)
*Soft tissue status*	
Open	5.60 (4)
Closed	94.40 (67)
*Subluxation at presentation*	
Sagittal	43.70 (31)
Coronal	52.10 (37)
*Acute immobilization*	
Temporary external fixation	5.60 (4)
Plaster immobilization	94.4 (67)
*Associated malleolar injury*	
Nil	2.80 (2)
Medial malleolus	83.10 (59)
Lateral malleolus	94.40 (67)
*Surgical fixation*	
Time awaiting surgical fixation (days)	19.06 (1–45)
Fragmentation	
Single fragment	71.80 (51)
2 part	22.50 (16)
≥ 3 part	5.60 (4)
*Fragment morphology*	
Comminution	53.50 (38)
Medial extension	28.20 (20)
Incisural extension	85.90 (61)
Sagittal fragment height (mm)	4.65 (3–37)
Sagittal percentage of AP fragment length^a,b,c,d^	12.55 (0.00–54.55)
Axial percentage of AP fragment length^a^	21.62 (4.37–50.00)
Sagittal percentage of surface length^b^	18.93 (0.00–57.49)
Axial percentage of perpendicular length**^c^	20.00 (4.17–48.78)
Percentage of axial fragment area^d^	9.56 (0.47–34.98)
Axial fragment area size (mm^2^)	124.41 (5.35–574.19)
Axial percentage of incisural fragment extension	19.30 ± 12.52
Primary fracture angle (°)	21.83 ± 11.36
Secondary fracture angle (°)	− 15.69 ± 13.80

Data are described as frequencies (counts), median (interquartile range) or mean ± standard deviation

*Including a fall from height (FFH), motor vehicle accident (MVA), blunt trauma and crushing injury

**A oblique measurement perpendicular to the main fragment fracture line

^a^Spearman Rank correlation *ρ* = 0.855, *p* < 0.001

^b^Spearman Rank correlation *ρ* = 0.625, *p* < 0.001

^c^Spearman Rank correlation *ρ* = 0.642, *p* < 0.001

^d^Spearman Rank correlation *ρ* = 0.595, *p* < 0.001

In 51 patients (71.8%), a single fragment was noted with a median axial area of 124.41 mm^2^ (IQR, 5.35—574.19), whilst 16 (22.5%) two-part and four (5.6%) three-part fragments were noted. The fragments were comminuted in 38 cases (53.5%) (Table 2).

Four methods were used to measure the posterior malleolus fragment’s AP length, and median values were used to compare central tendencies. Sagittal fragment measurements included the most commonly measured direct AP length of 12.5% (IQR, 0–54.5) and joint surface length of 18.9% (IQR, 0–57.5) of the plafond. On axial images, the AP length was 21.62% (IQR, 4.37–50.0), and the perpendicular length to the fracture line was 20% (IQR, 4.2–48.8) of the plafond. The fragment size as a percentage of the axial area was 9.6% (IQR, 0.47—34.9). A strong correlation in the sagittal plane between the AP length and surface length was observed (*ρ* [rho] = 0.855, *p* < 0.001), whilst moderate correlations were observed between the sagittal AP length and both the axial plane AP length (ρ [rho] = 0.642, *p* < 0.001) and perpendicular length (*ρ* [rho] = 0.595, *p* < 0.001), respectively. Moderate correlation was observed in the fragment size when comparing the sagittal AP length and the percentage area of the fragment in the axial plane (*ρ* [rho] = 0.595, *p* < 0.001). The primary fracture fragment angle from the trans-malleolar line was 21.83 ± 11.36 degrees (IQR, 0.1–52.3), with secondary fracture angles in multi-fragmented fractures of −15.69 ± 13.80 degrees (IQR, −48.2–4.9).

**Fig. 3:**
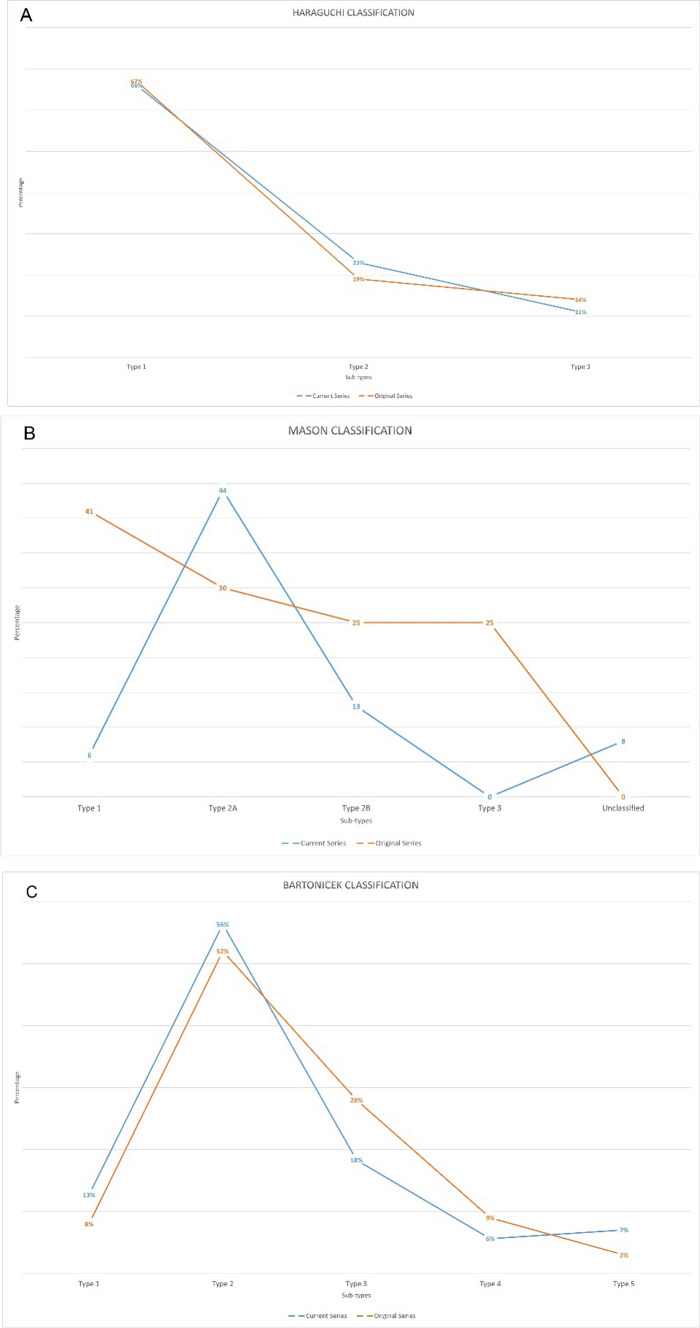
Classification correlation between our posterior malleolus fracture study cohort (blue) and the Haraguchi (**A**), Mason (**B**) and Bartonicek (**C**) original classifications (orange). Figure 3A Comparison between our posterior malleolus fracture patient cohort and Haraguchi et al. Figure 3B Comparison between our posterior malleolus fracture patient cohort and Mason et al. Figure 3C Comparison between our posterior malleolus fracture patient cohort and Bartonicek et al. (color figure online)

When comparing the established objective control classification for each classification system, no significant differences were noted for the Bartonicek (*p* = 0.221) and Haraguchi (*p* = 839) classifications. However, there were significant differences when comparing the data to the Mason classification (global *p* < 0.001) (Fig. 3).

 No agreement between reviewers for the Haraguchi (Krippendorf *α* = 0.560), Bartonicek (Krippendorf *α* = 0.506) or Mason (Krippendorf *α* = 0.557) classification systems was observed, with Krippendorff *α*-coefficients of less than 0.667 for all three systems (Table 3). When comparing the interobserver agreement, only 54% (*n* = 38) of Haraguchi classification assessments were unanimous across all observers. This was even lower at 46% (*n* = 32) for Mason’s classification and 42% (*n* = 30) for Bartonicek’s classification (Table 3). However, in patients whose classification type was unanimous, this correlated to the control classification for all Haraguchi types (38/38, 100%) and most Bartonicek (26/30, 87%) and Mason Classifications (27/32, 84%). The Krippendorff agreement test showed an *α*-coefficient value below 0.667 for all measurements, indicating no agreement between the observers (Table 3).

**Table 3:** Classification accuracy between five observers across three different CT classification systems for posterior malleolus fractures

Classification	Unanimous agreement between observers (*N* = 71)	Krippendorff α coefficient
Haraguchi	54% (38)	0.560
Bartonicek	42% (30)	0.506
Mason	46% (32)	0.557

Data are described as percentage of unanimous observer agreement with the total number of agreed cases compared in parentheses. The Krippendorff alpha coefficient includes multiple agreement variables and represents an overall agreement rate. It is interpreted as: < 0.667 indicating no agreement; 0.667–0.8 indicating tentative agreement; > 0.8 indicating high level of agreement as per the definitions of Krippendorff et al.

Sixty-six patients (93%) received operative fixation, and five (7%) were managed non-operatively (Table 4). All fracture types had varied methods of fixation used, with only Bartonicek type 4 fractures always being treated using a posterior tibial plate. No discernible pattern of fixation was identified across the classification types.

**Table 4:** Methods of fixation of all patients that underwent operative management for posterior malleolus fractures over a one-year period

Fixation	*N* = 66
No posterior malleolar fixation	5.6 (4)
Posterior tibial plate only	57.6 (38)
AP screw only	3.0 (2)
Syndesmosis screw only	21.2 (14)
Posterior tibial plate and syndesmosis screw	12.1 (8)

Data are described as frequencies with counts in parentheses. Four patients were not managed operatively (C-type host, declining surgery, inoperable soft tissue, prolonged delay to surgery), and a single patient was managed conservatively

## Discussion

Jehlicka et al. noted a bimodal distribution of PMF, with younger males in their 3rd decade and older, likely osteoporotic, females in their 6th decade incurring injury [4]. Additionally, multiple studies note a mean age range of 45–49 years [3–5, 19, 20, 34], with Odak et al., in a systematic review of 960 patients finding a 56.8% (*n* = 288/506) overall female predominance [1]. Our younger (40.8 years) female predominant (69%) cohort, with high (64.8%) unemployment rates, therefore depicts a unique population group compared to international literature.

Xu et al. in a high-volume study, observed that PMF’s are typically low-velocity injuries, resulting from a fall or stumble, and rarely due to high mechanisms of injury such as falls from height or road traffic accidents, contrary to what was found in this study, with nearly a quarter of all PMFs were due to high-velocity pedestrian-vehicle accidents [12, 34]. Considering the prevalence of these higher energy injuries, it is unsurprising that joint subluxation was noted in almost half (43.7% sagittal subluxation; 52.1% coronal subluxation) with a subsequent increased rate of post-traumatic arthritis expected [35, 36]. Thus, our higher energy, subluxated injuries pose a significant risk to poor outcomes and residual functional limitations in our population as an independent pathomechanical variable. Additionally, when comparing fracture patterns and fragment numbers to morphological studies, with no differences noted [18]. However, 53.5% of the injuries in the current series showed some degree of comminution. This is likely due to pathomechanical variables, and whilst no comparisons are available in the literature, this is thought to be high.

The current series measured the size of the posterior malleolus by four means: direct AP (sagittal), along the joint surface (sagittal), perpendicular to the trans-malleolar line (axial), and perpendicular to the fracture line (axial). Variations in fragment size were noted, however all studies showed variations in mean fragment size (range 16 – 25%), the current series appears consistent with prior studies.

The Bartonicek, Haraguchi and Mason classifications are the most well-known CT-based classifications, and typically used in clinical practice [9, 18, 19]. The authors’ individually described parameters were used to establish an objective control classification type for each fracture within each classification. It is not known if this method has been used to obtain an objective classification type, but we believe this form of measured assessment to be more accurate than observation alone. When the control classifications of the current cohort were compared to cited classifications, there was no significant difference between the current series and those of Bartonicek et al. or Haraguchi et al. The current series showed similar numbers and fracture patterns to studies conducted in predominantly European and Asian populations. The Mason classification, however, was dissimilar due to very few fractures in the present study having a pure coronal fracture line. This made a large proportion of injuries unclassifiable, according to the Mason classification. Several authors associated varying fracture types related to a specific mechanism of injury [9, 19]. Hence, the agreement of our population with that of Bartonicek’s is surprising, with a greater proportion of type four (axial compression) injuries anticipated. Whilst the exact position of the foot in relation to the tibia, the amount of rotatory energy transferred across the tibiotalar joint and the direction of force being unknown in pedestrian-vehicle accidents, a rotatory talar moment must still predominate, leading to similar fracture morphologies.

When comparing the observations of the five clinicians, unanimous agreement was seen in less than half of the cases (range 42–52%), with interrater agreement rated as “poor” for all the classification systems. Subsequent comparison to the control classification revealed that when observers were unanimously agreeable, there was a high accuracy and likelihood that they were correct in their assessment (range 84–100%). Furthermore, whilst still poor, the Haraguchi classification had the highest agreeability rates, likely due to it being the simplest classification, with distinct, clinically discernible variables and no size or percentage-based classification type variations. It is thus reasonable to state that whilst a correct diagnosis is possible, at acceptable rates, this is only possible to achieve once a unanimous diagnosis is made. In contrast to recent literature which notes substantial reliability and agreement to all three of the commonly utilized classification systems, our study observed “poor” interobserver reliability [37, 38]. The general poor agreement may be due to the complexity of the classifications themselves, clinical difficulties in recognizing these variables, or both. The need for clinical observations to be reliable requires no justification, as misdiagnosis leads to inconsistent treatment, as seen in this study, and ultimately poorer anticipated outcomes.

In recent literature, the concept of tibiotalar congruity, incisural reduction, syndesmotic stability and restoration of the posterior buttress is generally accepted as treatment goals [2, 12]. However, recent literature, including systematic reviews, found no consensus regarding the fragment size as an isolated threshold for surgical fixation with ankle joint stability, irrespective of fragment size gaining favour and well supported in literature [1, 11, 20, 21, 39, 40]. Whilst Gardner et al. found this to be the most significant surgical indicator, this opinion was only elicited in 56% of surgeons [13]. A standardized surgical indication or morphological threshold is not available at present. Subsequently, there are significant inconsistencies in treatment strategies.

In the current series, 93% of all PMFs underwent surgical fixation. Our disproportionately high rate of surgical intervention, with mean smaller fracture fragment sizes, indicates that we are potentially overtreating our patients. Some literature support fixation for most PMFs; however, the lack of consensus is evident in the high fixation rates noted in the current series. The fixation strategies observed in our study varied, with no identifiable predilection of one fixation method with type 4 Bartonicek fractures the only fracture pattern exclusively managed with posterior buttress plating. The lack of clear surgical indications and established treatment algorithms has resulted in the management of these injuries being highly inconsistent and an area of concern.

Investigating the short-and long-term outcomes of the patients included in this study was beyond the scope of the investigation, however, whilst not measured—they are thought to be sub-optimal–significantly affecting a unique demographic, which is a crucial area requiring further study in future.

## Conclusion

This study identified a unique, heterogeneous, at-risk, vulnerable population group in a resource-limited health care system. These patients are incurring higher mechanism injuries, high rates of subluxation at presentation and greater fragment comminution with representative fracture patterns to international literature. Despite this, they are poorly and unreliably classified. The lack of well-established treatment standardization, algorithms or consensus has resulted in sub-optimal management strategies and likely sub-optimal outcomes, which require further study.
